# Case Series Report: Reconstruction of Chronic Achilles Tendon Rupture With Semitendinosus Autograft Combined With Vulpius Technique

**DOI:** 10.7759/cureus.32325

**Published:** 2022-12-08

**Authors:** Mihail Vuldzhev, Jerome Linkwinstar, Filip Koynarski

**Affiliations:** 1 Orthopaedic Surgery, St. Sofia General Hospital, Sofia, BGR

**Keywords:** vulpius technique, lengthening, semitendinosus graft, chronic rupture, achilles tendon

## Abstract

We present five patients with chronic ruptures of the Achilles tendon and a defect larger than 6 cm. The average age of patients was 47 years. The interval between the rupture and treatment was between two and seven months. The size of the defects was between 6 and 12 cm, determined by magnetic resonance imaging (MRI) and/or ultrasound. All patients had an inability to step on the patient's limbs, a positive Thompson's symptom, difficulty walking and calf atrophy. Here, we evaluated the surgical results of semitendinosus autograft combined with Vulpius lengthening of the gastrocnemius in the treatment of chronic Achilles tendon rupture with a defect larger than 6 cm during medium-term follow‐up.

## Introduction

The Achilles tendon is the largest and strongest tendon in the human body. It is made up of the fusion of the tendons of the two heads of the gastrocnemius muscle, which occupy a superficial position, and the tendon of the soleus muscle, which occupies the deep layer. The deep fibers attach to the proximal part of the calcaneus, and the superficial ones continue distally and form part of the plantar fascia [1]. Its function is related to the plantar flexion of the foot and is extremely important for proper gait. Achilles tendon ruptures are more common in young athletes and also in the elderly which are not related to sports. Chronic ruptures are extremely rare, as easy diagnosis and a clear clinical picture usually lead to timely recognition of acute ruptures [2]. If the interval between injury and treatment is more than four weeks, the rupture is defined as chronic [3]. In these cases, the calf is progressively shortened. Active single-leg heel rise is not possible on the affected side. The push-off phase of the gait is also disturbed [4].

Treating fresh tears is usually not difficult but choosing the right surgical technique for chronic ones is a challenge. They require reconstructive procedures to fill the defect. There is an increased risk of skin complications such as non-healing wounds and infection [5]. There are a number of methods, the most commonly used being V‐Y tendon plasty [6], gastrocnemius fascial turndown flap [7,8], tendon transfer [9-11], allograft reconstruction [12], autograft reconstruction [10,11], synthetic graft augmentation [13,14], and biologic matrix augmentation [15]. There is no conventional therapy for chronic Achilles tendon rupture, particularly when the damage is larger than 6cm. Fibroadipose scar tissue can sometimes bridge the gap in chronic Achilles tendon rupture. Fibroadipose scar tissue is not as functional as a normal tendon and can cause ankle weakness and gait difficulties; thus, the scar tissue must be fully removed [16].

Tendon transfers have been widely used with good results, but in some patients, the loss of function of the transferred tendon may be symptomatic. After flexor hallucis longus (FHL) transfer, patients report loss of strength when flexing the first metatarsal and decreased strength when pushing off the foot. This is often not a problem for less active patients, but for those wishing to return to sports, a different approach is recommended. The use of semitendinosus reconstruction in such cases is supported by the literature. Maffulli et al. [17] administered minimally invasive reconstruction with semitendinosus in 26 patients with a mean follow-up of 8.2 years. In all, the circumference of the lower leg has increased significantly compared to preoperative. All patients returned to their previous employment with 22 returning to normal physical activity.

Our proposed algorithm for reconstruction with semitendinosus graft includes a minimally invasive inverted V-shaped gastrocnemius lengthening of the Achilles tendon - Vulpius procedure. The aim is to reduce the size of the defect as much as possible. This allows for multiple graft passes and a larger diameter of the reconstruction, similar to the native size of the Achilles tendon. From a study in the literature, we could not find a publication of that combination.

## Case presentation

Patient information and selection

In our hospital, St. Sofia General Hospital, between January 2017 and May 2022, five patients underwent semitendinosus autograft with a V-shaped gastrocnemius fascia lengthening for a chronic Achilles tendon rupture. They were between the ages of 20-60. The Thompson test was used to examine the Achilles tendon rupture and MRI and ultrasound were used to evaluate the type and size of the rupture.

All patients had given informed written consent that was in accordance with our institution's guidelines as well as the Declaration of Helsinki and Good Clinical Practice. All five patients were admitted to the hospital for a minimum of three days as this was a fixed regulation set by the national health insurance and no patients exceeded this hospital stay count.

Inclusion Criteria

This includes Achilles tendon rupture diagnosed more than four weeks after injury, patients with Achilles tendon rupture had undergone reconstruction with semitendinosus autograft with a V-shaped lengthening of the gastrocnemius fascia.

Exclusion Criteria

This includes acute Achilles tendon rupture, concurrent illnesses including local infection, and neurovascular damage.

Surgical technique

**Figure 1:**
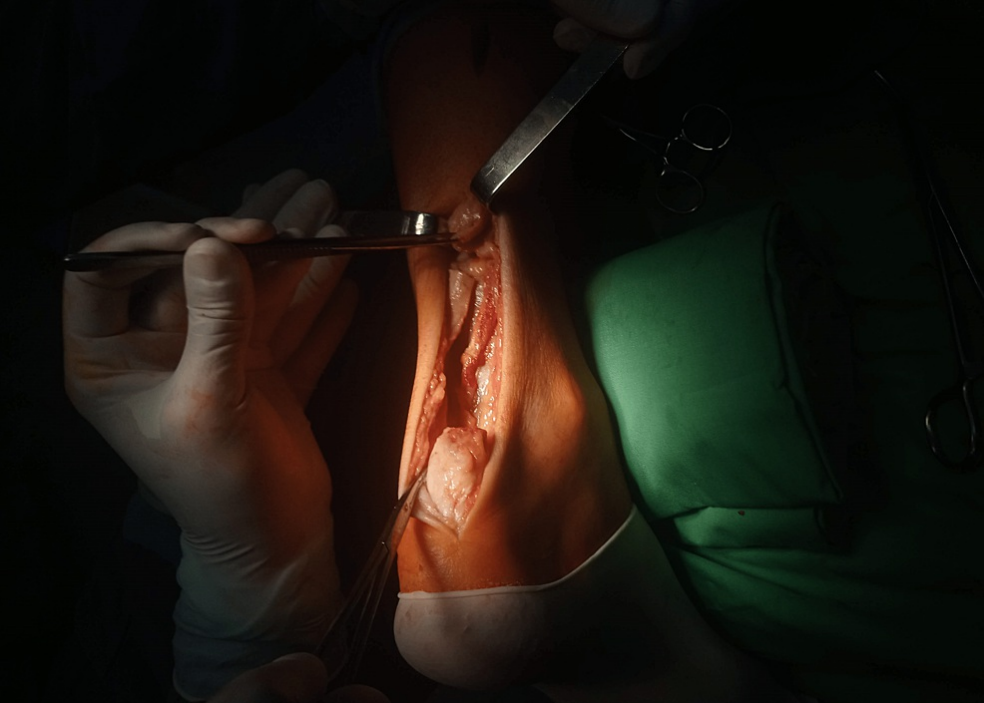
Intraoperative view after blunt dissection

The patient was positioned prone on the surgical table under lumbar plexus sciatic nerve block anesthesia with a tourniquet. The operative field was draped so as to provide access to both the Achilles tendon and the popliteal fossa. Standard posteromedial access to the Achilles tendon was performed. After dissecting the ends of the tendon, they were refreshed and the proximal stump is temporarily sutured (Figure 1). In a blunt way, the tendon is mobilized with a finger dissection and mobility is assessed. The tendon is usually rigid and has a large residual defect.

With additional proximal access, an inverted V-shaped extension of the gastrocnemius aponeurosis in the musculotendinous junction was performed. Once again, mobility was assessed by pulling the traction sutures, at the same time performing plantar flexion in the ankle joint and flexion in the knee joint.

**Figure 2:**
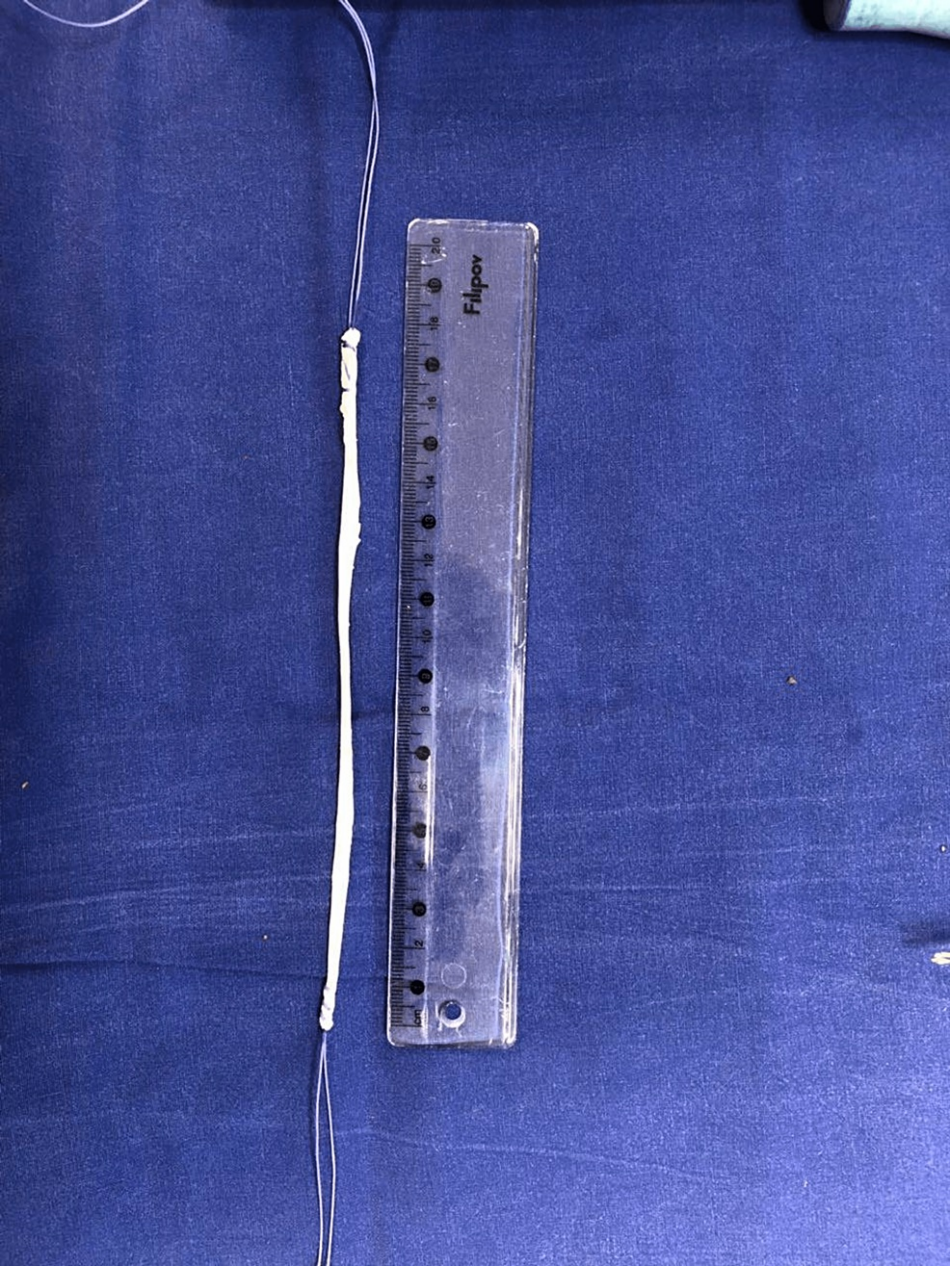
Length of semitendinosus graft

One free graft of the semitendinosus tendon was used to bridge the gap of the defect (Figure 2). Due to the fact that the patient was in a supine position, it is most convenient to take the tendon with small access in the area of ​​the popliteal fossa. The tendons of semitendinosus and gracilis were palpated and a cross-section of about 2 cm was made on them along the flexion fold, which gives the best cosmetic result. The semitendinosus tendon was dissected with a small swab and removed with an open stripper, first proximal, then distal. The resulting graft was sutured at both ends.

**Figure 3:**
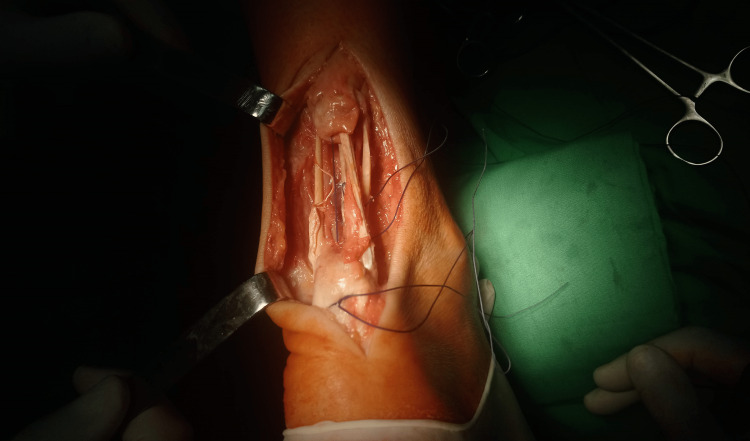
Single semitendinosus graft passed through stumps four times bridging the defect

With the help of a puncture needle and a mosquito hemostat, transverse tunnels were formed in the proximal and distal stumps of the Achilles tendon. With a sufficient length of the graft, it passes four times through the defect, and its edges were sutured (Figure 3).

**Figure 4:**
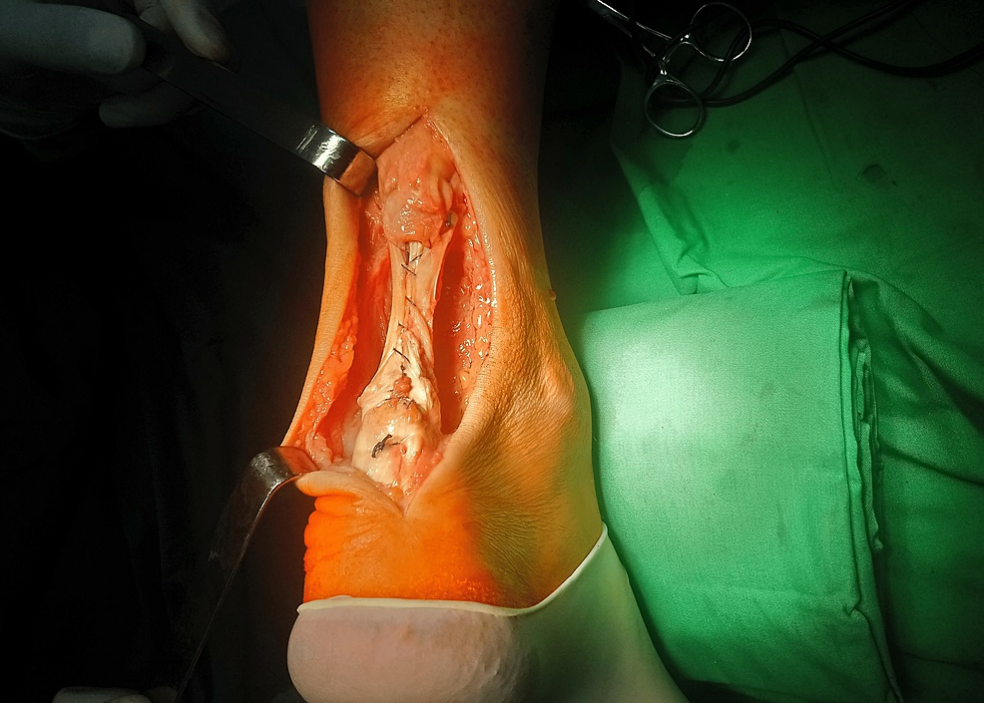
Final view of reconstruction after augmentation with Semitendinosus tendon

The graft was tabulated with additional threads (Figure 4). The ankle was placed in an adjustable boot in the position of maximum plantar flexion.

Postoperative treatment

The patients wore an adjustable boot for 10 weeks and walked with two crutches for six weeks. For the first four weeks, the boot was placed in the position of maximum plantar flexion. Each following week the dorsal flexion was increased by 10 degrees. After week 3, it was recommended to remove the immobilization in a controlled environment and electrical stimulation of the triceps and range of motion exercises were performed twice a week. Toe-touch weight bearing is allowed with slow progression. After six weeks, full weight bearing of the operated limb with protection from the boot is allowed. After the 10th week, the boot was removed, and the patients were encouraged to do active exercises for triceps muscle strength and ankle range of motion. Explosive activities were prohibited for six months - running, jumping, etc.

Results

**Figure 5:**
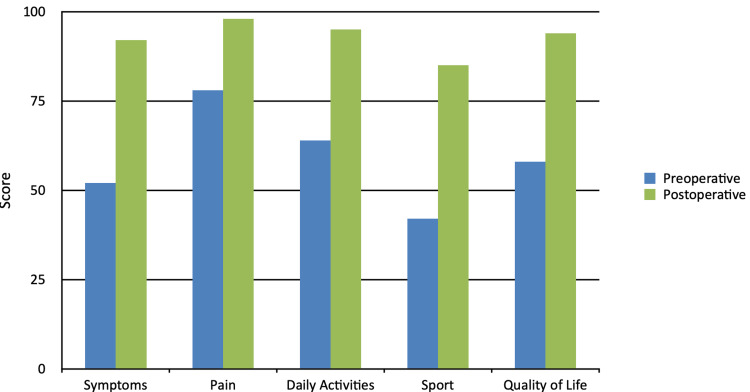
Clinical outcomes for subcategories

The results were reported according to the Foot and Ankle Outcome Score (FAOS), which has the following five subcategories - symptoms, pain, daily activities, sports, and quality of life [18]. The mean follow-up period was 2.6 years (range, 1-5 years). The mean outcomes of the subcategories of the scale were the following: symptoms 52 to 92, pain 78 to 98, daily activities 64 to 95, sports 42 to 85, quality of life 58 to 94, all preoperatively to postoperatively, respectively (Figure 5). The range for the ruptured gap was between 6 and 12 cm with a mean of 8.2 cm. The mean FAOS increased from 58.8 preoperatively to 92.8 during the follow-up period. There was an increase in the circumference of the calf by a mean of 1.4 cm (37.4 cm preoperative mean and 38.8 cm postoperative mean) when the follow-up period was over. There was still present atrophy compared to the circumference of the contralateral side, but with significant improvement. It had decreased from 14.3% to 5.6%. Patients returned to normal activity within the range of 5-9 months with a mean of 7.2 months.

During the follow-up period, no patients experienced a recurrence of the Achilles tendon rupture. No major problems, such as sural nerve damage or deep vein thrombosis had occurred in any of the patients. Superficial infection was observed in one of the patients who was successfully treated topically with dressings containing silver ions for two months in an outpatient setting. The five patients returned to their previous physical activity and one of them included amateur football.

## Discussion

In our real-life study, restoration of the chronic Achilles tendon ruptures using a semitendinosus autograft combined with the Vulpius technique resulted in tendon healing and satisfactory clinical and functional outcomes at a medium-term follow-up. In three months, all of our patients had resumed their regular activity levels from before their injuries. There was no functional impairment, and all patients were satisfied with the outcomes.

The most effective treatment for chronic Achilles rupture remains undecided. Although there are many [5-15], each method comes with its own benefits and drawbacks. There is no concrete guideline for treatment with a defect larger than 6 cm for a derelict Achilles tendon rupture. The semitendinosus autograft technique described by Patil et al. [19] reported satisfactory functional results. It is an economical method as suture anchors or tenodesis screws are not utilized.

The FHL transfer has evolved into an effective therapy for chronic Achilles tendon rupture. However, because this approach may impair halluce’s function, it should not be used on young patients regularly. Patients who were treated by FHL transfer, according to Wegrzyn et al. [20], engaged in fewer sports than before the injury. Furthermore, the interface structure repair between bone and tendon would be a lengthy procedure. Wegrzyn et al. found that patients treated with FHL transfer were able to go back to athletic activities after an average of 10 months [20].

Because chronic Achilles tendon rupture is uncommon, our investigation was hampered by a small patient population. Future research with a greater number of cases and a longer follow-up period may give more evidence for the utility of semitendinosus autograft together with the Vulpius technique in the treatment of chronic Achilles tendon rupture.

## Conclusions

The treatment of chronic Achilles tendon ruptures still remains controversial. Data from clinical trials clearly indicate better clinical outcomes in surgical treatment compared to conservative treatment. However, the ideal operational technique remains unclear. There are many techniques, each with its own advantages and disadvantages. Our preferred technique with Vulpius lengthening the gastrocnemius aponeurosis and filling the residual defect with free semitendinosus autograft gives good clinical results comparable to those in the literature for the treatment of chronic Achilles tendon. Furthermore, by employing an autograft rather than an allograft, this approach helps to reduce the additional dangers of an immune response or rejection associated with the use of allografts.
